# Influence of general practice opening hours on delay in seeking medical attention after transient ischaemic attack (TIA) and minor stroke: prospective population based study

**DOI:** 10.1136/bmj.a1569

**Published:** 2008-09-18

**Authors:** Daniel S Lasserson, Arvind Chandratheva, Matthew F Giles, David Mant, Peter M Rothwell

**Affiliations:** 1 Department of Primary Health Care, University of Oxford, Headington, Oxford OX3 7LF; 2 Department of Clinical Neurology, Stroke Prevention Research Unit, University of Oxford, John Radcliffe Hospital, Headington, Oxford

## Abstract

**Objective** To assess the influence of general practice opening hours on healthcare seeking behaviour after transient ischaemic attack (TIA) and minor stroke and feasibility of clinical assessment within 24 hours of symptom onset.

**Design** Population based prospective incidence study (Oxford vascular study).

**Setting** Nine general practices in Oxfordshire.

**Participants** 91 000 patients followed from 1 April 2002 to 31 March 2006.

**Main outcome measures** Events that occurred overnight and at weekends (out of hours) and events that occurred during surgery hours.

**Results** Among 359 patients with TIA and 434 with minor stroke, the median (interquartile range) time to call a general practitioner after an event during surgery hours was 4.0 (1.0-45.5) hours, and 68% of patients with events during surgery hours called within 24 hours of onset of symptoms. Median (interquartile range) time to call a general practitioner after events out of hours was 24.8 (9.0-54.5) hours for patients who waited to contact their registered practice compared with 1.0 (0.3-2.6) hour in those who used an emergency general practitioner service (P<0.001). In patients with events out of hours who waited to see their own general practitioner, seeking attention within 24 hours was considerably less likely for events at weekends than weekdays (odds ratio 0.10, 95% confidence interval 0.05 to 0.21): 70% with events Monday to Friday, 33% on Sundays, and none on Saturdays. Thirteen patients who had events out of hours and did not seek emergency care had a recurrent stroke before they sought medical attention. A primary care centre open 8 am-8 pm seven days a week would have offered cover to 73 patients who waited until surgery hours to call their general practitioner, reducing median delay from 50.1 hours to 4.0 hours in that group and increasing those calling within 24 hours from 34% to 68%.

**Conclusions** General practitioners’ opening hours influence patients’ healthcare seeking behaviour after TIA and minor stroke. Current opening hours can increase delay in assessment. Improved access to primary care and public education about the need for emergency care are required if the relevant targets in the national stroke strategy are to be met.

## Introduction

Transient ischaemic attack (TIA) and minor stroke are medical emergencies associated with a high risk of early recurrent stroke.^1^ ^2^ ^3^ ^4^ Recent studies have shown that prompt assessment and treatment after TIA and minor stroke can substantially reduce the risk of early recurrent stroke.^5^ ^6^ The Department of Health’s national stroke strategy^7^ and guidance from the National Institute for Health and Clinical Excellence (NICE)^8^ state that high risk patients must be seen within 24 hours after onset of symptoms. To improve access to health care for patients with TIA and minor stroke we need data on delays in seeking care and on the relative use of primary and secondary care emergency services. Previous research on behaviour after TIA has shown considerable variation in delay from the onset of symptoms to calling for help.^9^

A new general medical services (GMS) contract for primary care was introduced in April 2004,^10^ whereby responsibility for patients’ care by general practitioners was reduced to office hours (8 am-6 30 pm, Monday to Friday). Recent proposed changes to the contract^11^ could improve access to primary care, with general practices opening in the evenings and at weekends, but the influence this will have on clinical outcomes is uncertain. Little research has been published on the association between general practitioners’ opening hours and patients’ healthcare seeking behaviour, particularly in emergencies.

Given the recommendations of the national stroke strategy^7^ and NICE guidance^8^ that patients with TIA or minor stroke be assessed and treated urgently, we examined the relation between practice opening hours and delay to access healthcare in a population based study of all TIA and minor strokes, irrespective of age, and also determined the possible effect of the introduction of the new contract.

## Methods

The Oxford vascular study (OXVASC) is a population based prospective study of all acute vascular events in 91 000 patients registered at nine general practices in Oxfordshire and is fully described elsewhere.^12^ We included in this analysis all patients with a first incident or recurrent definite or probable TIA or minor stroke during the period 1 April 2002 to 31 March 2006. A study neurologist saw patients with TIA and stroke soon after the event, if possible within 24 hours. A consultant neurologist (PMR) reviewed all cases. TIA was defined as a focal neurological deficit lasting <24 hours and minor stroke defined as persisting deficit with National Institute of Health (NIH) stroke scale^13^ score <5 at the time of assessment.

We recorded the time of onset of the presenting event, time to calling healthcare services, and the choice of healthcare provider, along with clinical and sociodemographic data. Patients were asked what they thought had caused their symptoms. In a minority of patients for whom we did not know precise timing—for example, in patients presenting late or with cognitive impairment— we derived timings from ambulance sheets, general practitioner referral letters, and consultation notes. If these data were uncertain and patients recalled the approximate time of day we imputed the modal call time for that part of the day.

For the new contract time period, we divided the week into surgery hours, defined as the times when contact can be made with a patient’s registered general practice (Monday to Friday 8 am-6 30 pm) and out of hours (times outside this range). For the old contract time period, Saturday morning (9 am to 12 noon) was also classified as during surgery hours.

We calculated median call times and analysed differences between groups using the Mann-Whitney U test with SPSS software. Scatter plots were drawn of delay in calling a general practitioner against time of event to show patterns of behaviour.

We assessed the potential impact of increasing primary care opening hours to 8 am-8 pm daily (as in the polyclinic model of primary care^14^) on reducing delay. The patients with out of hours events that would become events during surgery hours with extended opening hours were assumed to behave in the same way as the overall surgery hours group. We estimated the potential impact on stroke prevention with daily 8 am-8 pm opening by analysing the subgroup of patients who did not seek attention after an initial TIA or minor stroke out of hours and went on to have an early recurrent stroke before their general practice reopened.

## Results

Of 359 patients with TIA and 434 with minor stroke, we excluded from the analysis 25 patients who were outside the study area at the time of their event. Mean (SD) age was 74.5 (11.8) years; 290 patients (38%) were aged ≥80; and 53% were women. Data on call delay were available in 721 patients (94%).

The table[Table tbl1] shows the numbers of patients choosing different healthcare providers for a first medical assessment after TIA or minor stroke. Most (73%) first sought attention from a general practitioner, with a small increase after introduction of the new GP contract in the numbers using accident and emergency departments (A&E) in patients with TIA (18% *v* 26%, P=0.055) but no change in those with minor stroke (23% *v* 24%, P=0.717). Some 387 patients had events during surgery hours, and 354 patients had events out of hours. For all patients in the out of hours setting, there was a non-significant increase in the proportion attending A&E after the introduction of the new contract (27% *v* 33%, P=0.216). Use of A&E was not influenced by correct recognition of symptoms or by age. Only 10 patients called NHS Direct, of whom seven were advised to be seen routinely in primary care and three to attend A&E.

**Table tbl1:** First healthcare provider after TIA or minor stroke, stratified by old and new general practitioner contract and by events during surgery hours or out of hours. Figures are numbers (percentages) of patients

	TIA			Minor stroke	
	Old	New		Old	New
Attending provider*:					
GP	147 (78.6)	114 (70.8)		159 (71.9)	141 (70.9)
A&E	33 (17.6)	42 (26.1)		50 (22.6)	48 (24.1)
Other clinic	4 (2.1)	4 (2.5)		2 (0.9)	0
Inpatient services	3 (1.6)	1 (0.6)		10 (4.5)	10 (5.0)
Event during surgery hours:					
GP	78 (84.8)	63 (71.6)		86 (75.4)	74 (79.6)
A&E	11 (12.0)	21 (23.9)		20 (17.5)	13 (14.0)
Other clinic	1 (1.1)	3 (3.4)		2 (1.8)	0
Inpatient services	2 (2.2)	1 (1.1)		6 (5.3)	6 (6.5)
Event out of hours:					
GP	63 (72.4)	49 (70.0)		68 (69.4)	64 (64.6)
A&E	21 (24.1)	21 (30.0)		28 (28.6)	34 (34.3)
Other clinic	2 (2.3)	0		0	0
Inpatient services	1 (1.1)	0		2 (2.0)	1 (1.0)

GP=general practitioner; A&E=accident and emergency department.

*Includes 27 patients for whom choice of provider was known but data on timing were missing.

There was no difference in median delay to call for help or in place of seeking medical attention in patients with a first ever incident event and patients with recurrent events. We therefore performed a pooled analysis.

The median (interquartile range) time to call a general practitioner was significantly greater in the out of hours setting compared with during surgery hours: 12.0 (2.1-43.0) *v* 4.0 (1.0-45.5) hours, P=0.006. Median (interquartile range) time to call for medical attention via A&E was not significantly different between events occurring out of hours and events during surgery hours: 0.91 (0.33-2.68) *v* 0.73 (0.38-2.01) hours, P=0.751.

Of 244 patients who had events out of hours and were seen first in primary care, 175 (72%) waited to call their registered practice, although under the new contract there was a significant increase in the percentage of patients who used an on-call general practitioner service (20% *v* 32%, P=0.034). Median (interquartile range) time to call a general practitioner was significantly higher in those who did not, compared with those who did, use an on-call general practitioner service (24.8 (9.0-54.5) hours *v* 1.0 (0.3-2.6) hours, P<0.001). Use of an on-call general practitioner service was unrelated to age or to correct recognition by the patient of the cause of their symptoms.

Figure 1 shows the number of patients with out of hours events who called within successive two hour time intervals after their registered practice opened. Most (70%) of the patients who waited to call their general practitioner did so within two hours of the practice opening. Figure 2 shows the delay to calling the registered practice after an out of hours event for the first 72 hours after events. As predicted, given that most patients call as soon as possible after the practice opens, in the overnight period on weekdays there was a linear relation between delay and the time of day of the event, with delay reducing as event time approaches practice opening hours. For most patients with events at the weekend, the delay was also related to proximity to earliest opening hours of the practice (that is, Monday morning) and patients with events on a Saturday waited longer to call for medical attention than patients with events on a Sunday. In patients with events out of hours who waited until practice opening hours to call, 70% with events on Monday to Friday rang within 24 hours of symptom onset, but this proportion fell to 33% on Sundays and none on Saturdays. The odds ratio of calling within 24 hours after weekend events compared with weekday events was 0.10 (95% confidence interval 0.05 to 0.21).

**Figure fig1:**
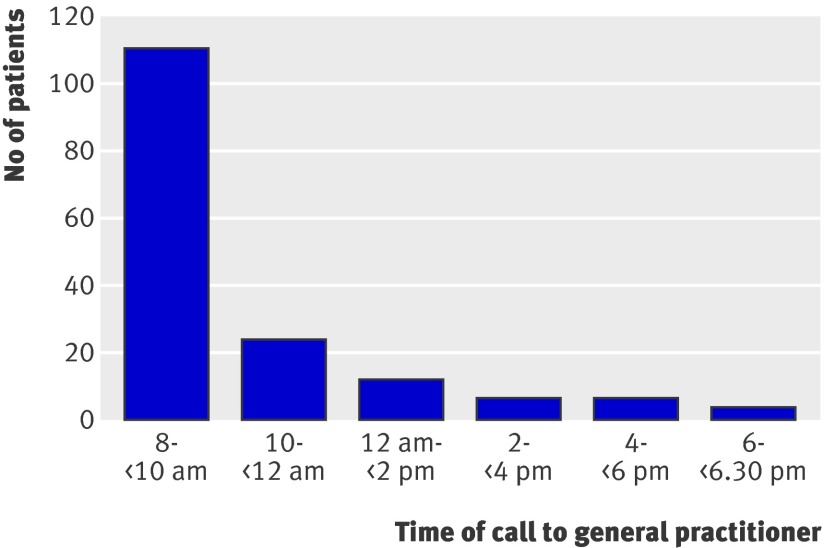
**Fig 1** Number of patients calling their registered practice after out of hours event by time of call

**Figure fig2:**
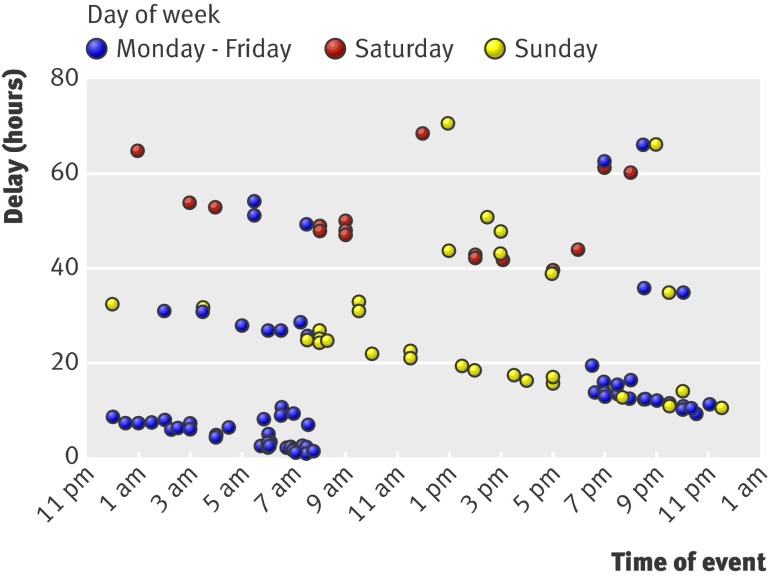
**Fig 2** Delay in calling regular general practitioner after TIA or minor stroke occurring out of hours

The plot of delay against time of event for patients with events in hours (fig 3) also shows clustering of delay. Some patients with events during surgery opening hours also seek care in the days after the event, with gaps on the scatter plot corresponding with times when the practice is closed. Around two thirds (68%) of patients with events during surgery hours called within 24 hours.

**Figure fig3:**
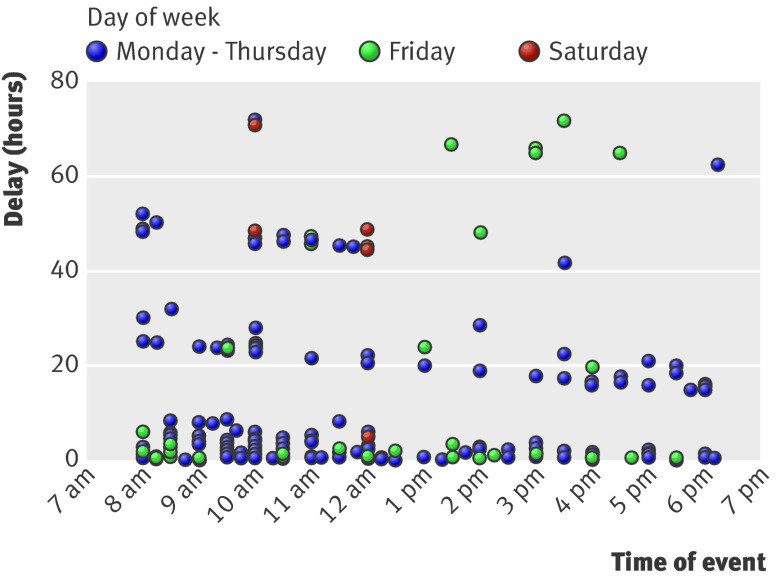
**Fig 3** Delay in calling general practitioner after TIA or minor stroke occurring during surgery hours

We analysed the possible effects of extended opening hours—for example, from 8 am to 8 pm seven days a week. Seventy three patients who waited until office hours to call (30% of the group with events out of hours seen in primary care) had events at times during the extra period of cover that such centres would offer. The median (IQR) delay to calling primary care in this subgroup was 50.1 (22.5-118.0) hours, and 34% called within 24 hours of symptoms. If these patients behaved like the patients with events during surgery hours then delay would be reduced to 4.0 (1.0-45.5) hours and the percentage calling within 24 hours would increase to 68%.

Increasing opening hours could reduce recurrent stroke. Thirty seven patients had a recurrent stroke after an initial TIA or minor stroke for which they did not seek medical attention. Of these, 13 had an initial event out of hours and five had an event at times during the period of extra cover that a centre open 8 am to 8 pm daily would offer. Thus five strokes occurred in patients for whom extending general practice opening might have allowed an opportunity for clinical assessment and treatment after TIA before a recurrent stroke.

## Discussion

Most patients chose to seek help from their own practice after TIA or minor stroke. The new general practitioner contract did not significantly increase the use of A&E outside office hours but small improvements were seen in the use of emergency primary care services. The few patients who used NHS Direct had variable guidance, with most advised to attend primary care routinely. Among patients seen in primary care, considerable delays in calling for medical attention were seen after events out of hours compared with events during surgery hours, and striking patterns of delay (figs 2 and 3) resulted from patients waiting for the earliest opportunity to contact their registered practice. A small number of patients who had an event out of hours delayed seeking care and went on to have a recurrent stroke before their practice reopened. Increased access to general practice out of hours could provide an opportunity for assessment and urgent referral.

### Strength and weaknesses

Our study was population based with high levels of case ascertainment, particularly of elderly patients. There are, however, some potential shortcomings. Firstly, we did not have exact data on timings of events and on delays to seeking medical attention in all patients because of factors such as dysphasia and cognitive impairment. Reliable data, however, were available in 94% of patients, of whom over 37% were aged ≥80, and so there is unlikely to have been substantial inclusion bias. Secondly, TIAs and minor strokes before major disabling strokes might have been under-reported as some patients with major stroke are unable to give an account of previous TIA or minor stroke and corroborative accounts are not always available. We could therefore have underestimated the number of strokes that might be prevented by greater access to primary care. 

Recognition of stroke-like symptoms was not associated with shorter delays to call for medical attention or with use of emergency services. Similar findings in other studies^15^ ^16^ ^17^ suggest a need for more public education, although awareness campaigns have not always had a predictable effect.^18^ There might be a future role for NHS Direct in reducing delays to assessment by acting as a coordinating service—for example, directing patients to a stroke unit for assessment as part of a comprehensive stroke and TIA care pathway—but few patients in our study were currently using this service and the advice given was not always optimal, which limits the options for the planning of integrated care.

Increasing access to primary care might have a variable impact on TIA and stroke outcomes. If general practitioners had access to urgent secondary care investigation and treatment with effects similar to those in the EXPRESS study of an 80% relative risk reduction in recurrent stroke,^5^ our data suggest that one stroke per 91 000 population per year could be prevented (that is, over 500 strokes annually in England alone). Even though most patients in the study sought help from primary care, however, this might not be the optimal model of service delivery. Longer delays from onset of TIA or stroke symptom to emergency hospital admission have consistently been associated with involving primary care rather than simply calling for an ambulance.^19^ ^20^ ^21^ ^22^ These studies were mainly of acute stroke and had few patients with TIA and consequently did not assess factors that increase delay in assessment in an outpatient based system of TIA care, which is the current UK model.

Furthermore, there are few studies on the accuracy of diagnosis of TIA in primary care or in A&E. Longer opening hours in primary care as well as increased direct attendance of unselected patients at A&E might increase the referral rate to specialist neurovascular services, although how much of this increase would be because of prompt referral of appropriate patients after onset of symptoms depends on the diagnostic specificity of the referring clinician.

### Relevance of findings

The opening hours of general practices seem to influence the behaviour of most patients with TIA and minor stroke occurring out of hours. The national stroke strategy^7^ sets targets for assessment that are a challenge across the services of the NHS, and there might not be one single approach that helps to meet these targets. Increased practice opening hours is one possibility but requires secondary care capacity in terms of access to investigations and specialist assessment if the full benefits in stroke prevention are to be seen.

The key questions for future research lie in the interface between patients and their health system. Qualitative studies might shed light on the reasons for patients’ choice of healthcare provider after TIA and minor stroke and the reasons for delay in seeking health care. This might also help in decisions about the content and delivery of effective public health awareness campaigns. Given that most patients are seen in primary care, we need to study the accuracy of general practitioners’ diagnosis of TIA and stroke. The small take up of NHS Direct for advice about when and where to seek care also requires investigation..

**What is already known on this topic**

Urgent treatment after TIA and minor stroke can prevent recurrent disabling or fatal stroke
The Department of Health’s national stroke strategy calls for investigation of high risk patients with TIA within 24 hours after onset of symptoms
Most patients with TIA or minor stroke seek health care via their general practice rather than via emergency services

**What this study adds**

After TIA or minor stroke, only 1% of patients contact NHS Direct for advice
Most patients with TIA and minor stroke out of hours delay seeking health care until their registered general practice is open, causing long delays, particularly at weekends
Patients with out of hours events are much less likely to call their GP within 24 hours after symptoms at weekends than overnight on weekdays

## Notes

Ethical approval: The Oxford vascular study was approved by the Oxfordshire clinical research ethics committee (CO.043).

Provenance and peer review: Not commissioned; externally peer reviewed.

## Notes

**Cite this as:** *BMJ* 2008;337:a1569
